# A new mutation in blau syndrome: case report

**DOI:** 10.1186/1546-0096-12-S1-P297

**Published:** 2014-09-17

**Authors:** Cengiz Zeybek, Davut Gül, Faysal Gök

**Affiliations:** 1 Pediatric Rheumatology, Genetics, Gülhane Military Medical Academy, Ankara, Turkey; 2 TBC

## Introduction

Blau syndrome is a rare autoinflamattory granulomatous disease and inherited as autosomal dominant.The classical triad of Blau syndrome is granulomatous dermatitis, symmetric arthritis and recurrent uveitis. However, all of these findings may not be together in the patients. In the majority of patients, the disease is characterized by early onset that usually before 3-4 years of age. The ocular findings of Blau syndrome ocur usuaaly after the articular and skin findings.

## Objectives

Our aim is to describe a new mutation of Blau syndrome here. The defective gen of Blau syndrome is located 16q12.2-13 locus and NOD2 gen is found in this locus. So far, ten NOD2 mutations have been described that causes Blau syndrome. In addition, seven NOD2 mutations have been described that may be associated with Blau syndrome. These mutations are heterozygous state mostly.

## Methods

Five years old male patient had developed papular rash that lasted one year at 5 month old, bilateral knee and ankle arthritis at 4 year old and right anterior uveitis at 5 year old. His papular rush and anterior uveitis was compatible with granulomatous vasculitis and granulomatous uveitis, respectively.

## Results

Blau syndrome gen studies revealed heterozygous missense NOD2 mutation ( P507S [(c.1519C>T)].

## Conclusion

Up to date, 10 Blau-associated genetic mutations have been identified within thisgene, almost in heterozygous state. Two of these mutations (R334Q and R334W) account for more than 50% of the mutated alleles. The mutation that we found is a new mutation and not described before.

## Disclosure of interest

None declared.

